# HOSPICE UTILIZATION AMONG MEDICAID-ONLY AND DUALLY ELIGIBLE DECEDENTS IN CONNECTICUT

**DOI:** 10.1093/geroni/igac059.162

**Published:** 2022-12-20

**Authors:** Julie Robison, Deborah Migneault, Noreen Shugrue, Doreek Charles, Bradley Richards, Dorothy Wakefield, Ellis Dillon, Dawn Lambert

**Affiliations:** UConn Health, Center on Aging, Farmington, Connecticut, United States; UCONN Health, Farmington, Connecticut, United States; University of Connecticut, Farmington, Connecticut, United States; University of Connecticut, Farmington, Connecticut, United States; State of Connecticut, Hartford, Connecticut, United States; University of Connecticut, Farmington, Connecticut, United States; University of Connecticut, Farmington, Connecticut, United States; State of Connecticut, Hartford, Connecticut, United States

## Abstract

Individuals with Medicaid as their health care payer source may be either “Medicaid-only,” or “dually eligible,” i.e., qualifying for both Medicaid and Medicare. Medicare included hospice as a full Medicare benefit in 1982; Hospice is an optional Medicaid benefit for states; Connecticut added the Medicaid hospice benefit in 2010. This study explores hospice use in Connecticut’s Medicaid program by comparing hospice use and length of hospice enrollment by Medicaid-only vs. dually eligible and by diagnosis for decedents who died between 2017 and 2020 and had a hospice-appropriate diagnosis. This analysis of 2,990 Medicaid-only and 24,881 dually eligible decedents finds that dually eligible decedents had a significantly (p<.001) higher rate of hospice use (48.1%) compared to Medicaid-only decedents (29.9%). Medicaid-only decedents received hospice for a median of 12 days vs. 13 days for the dually eligible (p=.12). Dually eligible decedents consistently received hospice more often than Medicaid-only decedents across all diagnoses (cancer (p<.05) circulatory (p<.001), dementia (p<.001), respiratory (p<.001) and stroke (p<.001)). Dually eligible decedents had significantly more days of hospice than Medicaid-only decedents for subgroups with a circulatory (p<.05) or stroke (p<.05) diagnosis, but Medicaid-only decedents with a dementia diagnosis had more days of hospice than their dually-eligible counterparts. Additional multi-variate analysis will demonstrate how other factors, like age, may account for differences. Recommendations include encouraging physician use of hospice screening tools and developing education interventions with physicians and patients to increase understanding of the availability and benefits of hospice with particular focus on Medicaid-only patients.

